# A control theoretic primer for systems endocrinology

**DOI:** 10.3389/fendo.2026.1933159

**Published:** 2026-09-04

**Authors:** Massoud Boroujerdi

**Affiliations:** Independent Researcher, London, United Kingdom

**Keywords:** bode plot, control theory, cortisol, feedback regulation, frequency-domain analysis, HPA axis, mathematical modelling, metabolic clearance rate

## Abstract

Endocrine systems are characterised by complex dynamic behaviours—oscillations, transient responses, feedback regulation, and noise filtering—that are essential to physiological stability. Despite extensive molecular and clinical research, the quantitative principles governing these dynamics remain incompletely understood. In particular, the mechanistic relationship between the metabolic clearance rates of individual hormones and emergent system-level properties such as oscillatory frequency, damping, and stability has not been systematically integrated into endocrine theory.

This paper introduces a control-theoretic framework for systems endocrinology, modelling endocrine networks as cascades of second-order negative feedback systems whose parameters are derived directly from measurable clearance-rate values. Two transfer function architectures are defined—G-type (feed-forward) and H-type (feedback)—which encode a spectrum from rapid, responsive regulation to stable, noise-rejecting maintenance. Frequency-domain analysis via Bode plots extracts biologically interpretable metrics including cutoff frequency, bandwidth, phase margin, and stability margins. The framework is demonstrated using the cortisol-HPA axis as a worked example, where model-predicted timescales are shown to be consistent with experimentally observed ultradian pulsatility (~90-minute period), ACTH-stimulation response kinetics, and dexamethasone suppression dynamics. A reference table of clearance-rate-derived parameters for 36 hormones and substrates is provided, enabling immediate application across major endocrine axes. All computational tools are implemented in R and made freely available. By translating standard pharmacokinetic data into the language of control engineering, the framework provides a candidate, testable account of questions that static endocrine models leave qualitative: why cortisol pulsatility occurs on an approximately ultradian timescale, how stable the HPA axis is, and why the timing—not just the dose—of cortisol replacement determines clinical outcome.

## Clinical context

A patient with primary adrenal insufficiency takes hydrocortisone every morning. Her cortisol levels are pharmacokinetically adequate—the area under the curve matches reference ranges—yet she remains fatigued and cognitively impaired. She is enrolled in a crossover trial comparing once-daily Plenadren with twice-daily Chronocort, which delivers its dose at 02:00 and 06:00 h rather than on waking. On Chronocort, her fatigue scores improve significantly. The total cortisol exposure is similar. The difference is timing.

Standard pharmacokinetic models describe exposure but do not, by themselves, characterize the frequencydomain properties of downstream endocrine signaling. currently there is no framework for explaining this. The PK/PD models describe how cortisol concentrations rise and fall, but not *which frequencies of cortisol input* the downstream receptor systems are tuned to receive. To explain why phase matters as much as amplitude—why delivering the right signal at the wrong time is equivalent, from the cell’s perspective, to delivering the wrong signal—we need a different kind of model.

This paper provides that model. It treats the HPA axis as a feedback control system whose dynamic properties are directly encoded in the metabolic clearance rates of its constituent hormones. The framework predicts not only that cortisol should pulse at approximately 90 minutes, but *why*—and it generates quantitative predictions about what goes wrong in clinical HPA dysregulation states and how replacement therapy can be rationally designed to restore the correct spectral content of cortisol signalling.

## Introduction

### The temporal dimension of endocrine regulation

Endocrine systems do not operate in static equilibrium. Hormonal signals are inherently dynamic: they pulse, oscillate, decay, overshoot, and integrate over characteristic timescales that are as physiologically important as their steady-state concentrations. The ultradian pulsatility of cortisol (~90-minute period), the circadian rhythm of melatonin, the rapid clearance of angiotensin II (~1-minute half-life), and the prolonged half-life of thyroxine (~7 days) all reflect a fundamental principle: the temporal behaviour of a hormone is determined by the interplay between its production kinetics and its metabolic clearance rate, filtered through the feedback architecture of the axis in which it operates (1).

Yet despite this dynamic complexity, most computational approaches to endocrine regulation treat hormonal systems as static or quasi-static, characterising steady-state concentrations and network topology without addressing how fast a system responds, how selectively it filters noise, or how stable it is in the face of perturbation. These are precisely the questions that control theory was developed to answer. A concrete illustration is provided by the clinical vignette above and discussed in detail in the Discussion: the superiority of phase-matched over amplitude-matched cortisol replacement in adrenal insufficiency is a direct, falsifiable prediction of the framework presented here.

### Control-theoretic concepts in brief

Because the terminology used throughout this paper—natural frequency, damping ratio, transfer function, Bode plot—will be unfamiliar to many endocrinologists, we introduce these concepts here in plain terms before applying them formally in the Methods.

A hormonal axis is a *dynamical system*: its output at any moment depends not only on the current input (e.g. a secretory pulse) but on the system’s own internal state—how far concentrations currently sit from their eventual resting values. The model developed here represents each hormonal element as two interacting compartments, *S* (the hormone or substrate itself) and *C* (a controller-like compartment that *S* drives and that in turn regulates the loss of *S*). Because the system is built from two coupled first-order equations, it is called *second-order*, and its behaviour is fully characterised by two numbers: a *natural frequency* (*ω_n_*) and a *damping ratio* (*ζ*). A useful mechanical analogy is a car’s suspension: push the body down and release it, and with a soft damper it bounces a few times before settling (*ζ* < 1, *underdamped*), while with a stiff damper it returns to rest without overshoot (*ζ ≥* 1, *critically* or *overdamped*). *ω_n_* describes how fast the system would oscillate with no damping at all; *ζ* describes how strongly that oscillatory tendency is suppressed. Both numbers govern the response whether or not any oscillation is actually visible in it—which is why a natural frequency can meaningfully be assigned even to a hormone whose plasma profile is reported simply as a monotonic decay with a single half-life. That half-life describes only the isolated behaviour of *S* in a simplified one-compartment view; once *S* is placed back inside its feedback loop with *C*, the *loop’s* combined dynamics—which is what *ω_n_* and *ζ* characterise—are a distinct and generally faster or better-damped property of the whole system, not of the hormone in isolation. The half-life-derived rate constant is simply the most readily available clinical estimate of the underlying clearance kinetics that feed into that loop.

This also motivates why *frequency-domain* analysis (Bode plots) is useful here even though the underlying endocrine biochemistry is nonlinear. Rather than asking how a system responds to one particular input, a Bode plot asks how it would respond to inputs varying at many different speeds, and summarises the answer as two curves: how strongly each input speed is amplified or attenuated (*gain*), and how much it is delayed (*phase*). This is the same principle used to characterise an audio filter or a car’s suspension without needing the underlying physics to be perfectly linear: the analysis is a local, linearised approximation valid around a physiological operating point, exactly analogous to how a single cr value is itself a linearised summary of underlying nonlinear elimination kinetics. Two frequency-domain quantities recur throughout: the *cutoff frequency* is the input speed beyond which the response falls off sharply, and *bandwidth* is the range of speeds the system tracks faithfully below that point. A hormone with high bandwidth follows fast physiological demands closely; one with low bandwidth smooths over rapid fluctuations but responds only sluggishly to genuine, sustained signals—the speed-versus-stability trade-off that motivates the G-type/H-type distinction developed below.

It is worth addressing directly a question the two-compartment structure naturally raises: if, under a constant input, *_C_*settles to a fixed steady-state value ( $\bar{C}=R_{a}/k_{3}$), in what sense is *C* acting as a *controller*? A constant input producing a constant output at every compartment, including *C*, is not a shortcoming of the model—it is what any stable regulator does once equilibrium is reached; a thermostat holding a room at a fixed temperature also settles to a fixed duty cycle. The regulatory action of *C* is visible not at that single equilibrium but in how the system responds when it is displaced from it: *C*’s coupling back into the loss term of *S* is precisely what shapes the transient response—its speed (*ω_n_*), its overshoot or lack thereof (*ζ*), and its rejection of high-frequency perturbations (the Bode plot’s high-frequency rolloff). In other words, *C*’s controlling role is a dynamic, not a steady-state, property, and it is exactly what the frequency-domain metrics developed in this paper are designed to quantify.

### Control theory and endocrine systems

Control theory—the mathematical framework for analysing feedback systems—has been applied to physiological problems since the 1960s, from Guyton’s cardiovascular models to more recent work on the HPA axis (2, 3), the HPT axis (4, 5), the glucose-insulin system (6), and reproductive endocrinology (7). A hallmark of control-theoretic analysis is frequency-domain characterisation via Bode plots, which reveals how a system amplifies or attenuates signals at different temporal frequencies—directly addressing questions of response speed, noise rejection, and oscillatory tendency that are central to endocrine physiology (1).

A key insight motivating this framework is that the clearance rate (cr) of a hormone—defined as the volume of plasma cleared per unit time, and routinely measured in clinical pharmacokinetics as the metabolic clearance rate—contains direct information about the system’s natural frequency and hence its temporal dynamics. The cr is not merely a pharmacokinetic descriptor; it is a fundamental determinant of how quickly a hormonal compartment responds to perturbation and how effectively it rejects high-frequency noise. By linking cr to the natural frequency (*ω_n_*) of a second-order feedback model, the full frequency-domain behaviour of any endocrine element can be derived from a single, clinically measurable parameter. For this derivation, the cr must be expressed in units of time*^−^*^1^; when the published value is in ml/min/kg, conversion requires a volume of distribution, for which the accessible plasma volume of 41.1 *±*5.6 mL/kg body weight can be used (8–10).

### The second-order negative feedback model

This paper builds on a previously validated second-order negative feedback compartmental model developed for plasma concentration of substrate or hormone kinetics (11, 12). The model captures the essential dynamics of a substrate-controller pair and has been parameterised using clinical pharmacokinetic data. The central extension introduced here is the systematic derivation of model parameters from published cr values, enabling application across the full breadth of the endocrine system without requiring bespoke parameter estimation for each axis.

### Objectives

This paper serves as a methodological primer for systems endocrinologists and mathematical modellers. Specifically, we aim to:

1. Present the second-order negative feedback framework and its transfer function formulations in a form accessible to endocrinologists without extensive control-engineering background.
2. Demonstrate how cr values map to natural frequency, damping ratio, and Bode plot characteristics with direct biological interpretation.
3. Illustrate the framework using the cortisol-HPA axis as a worked example, showing consistency with known experimental observations.
4. Provide a reference parameter table for 36 hormones and metabolic substrates to enable immediate application.
5. Describe the open-source R implementation and validation approach.

## Methods

### Mathematical framework

#### Second-Order Negative Feedback Model

The fundamental building block of the network model is the second-order negative feedback compartmental system (11, 12). Each hormonal element is represented by two compartments: *_S_*(substrate or hormone) and *C* (controller), governed by:


$$\frac{dS}{dt}=R_{a}-k_{3}C$$

$$\frac{dC}{dt}=k_{4}S-k_{2}C$$

At steady state, the production rate relates to concentration and vd (volume of distribution) via the cr which is clearance rate:


$$R_{a}=\frac{k_{3}k_{4}}{k_{2}}S=\text{cr}\cdot S$$

where cr = *k*_3_*k*_4_*/k*_2_ with dimensions of time*^−^*^1^. The cr is thus directly embedded in the model structure. In situations where the cr value has been measured as ml/min/kg, a value with units of time*^−^*^1^ can be calculated by assuming a volume of distribution for *S* as accessible plasma volume of 41.1 *±* 5.6 mL/kg. In many other situations the dynamics of hormones and substrates are simply quoted as half-life. In such cases, using a one-compartment approximation, the half-life can be transformed to a rate constant as: *k* = ln(2)*/t*_1_*_/_*_2_. This rate constant can then be used as a measure for cr. The state-space representation is:


$$\dot{x}=Ax+Bu,\;y=Cx$$

where:


$$A=[\begin{array}{cc} 0 & -k_{3}\\ k_{4} & -k_{2} \end{array}],\;B=[\begin{array}{c} 1\\ 0 \end{array}]$$

A numerical simulation of the above system is presented as an R Shiny app available at https://github.com/mboroujerdi/servo-shiny-app. Github.

#### Transfer function parameters

The system parameters relate to the standard second-order differential equation as follows:

- Natural frequency: $\omega_{n}=\sqrt{k_{3}k_{4}}$
- Damping ratio: $\zeta =k_{2}/(2\omega_{n})$
- Equivalently: $k_{2}=2\zeta \omega_{n}$ and $k_{3}=k_{4}=\omega_{n}$G-Type and H-type transfer functions

Two transfer function architectures are defined. Before the mathematical definitions, **Table 1** grounds the distinction in named clinical examples. The key principle is that the architecture follows from the biological role: hormones in acute regulatory cascades (where response speed matters more than stability) use G-type; hormones in maintenance regulation (where stability and noise rejection matter more than speed) use H-type. The cr itself is the primary determinant—fast clearance implies high *ω_n_*and G-type assignment; slow clearance implies low *ω_n_* and H-type assignment.

**Table 1:** G-type and H-type transfer function architectures: clinical anchor examples.

Architecture	Transfer function	Clinical example	Biological role
G-type (feed-forward)	Includes phase-lead zero; faster response	ACTH (half-life ~10 min)	Rapid acute signalling; stress response
H-type (feedback)	Pure lag; stronger noise rejection	Cortisol (half-life ~90 min)	Stable maintenance; negative feedback set-point

Architecture assignment is determined by the clearance rate (cr): fast clearance (high *ω_n_*) yields G-type; slow clearance (low *ω_n_*) yields H-type.

G-type _(feed-forward):_Uses the *k*_3_*C*flux. This architecture is suitable for situations where a hormone is directly secreted into the circulation.


$$G_{i}(s)=\frac{k_{3i}(s+k_{2i})}{s(s+k_{2i})+k_{3i}k_{4i}}$$

This includes a zero at *s* = *−k*_2_*_i_*, providing phase lead compensation and faster response. Appropriate for hormones in acute regulatory cascades.

H-type (feedback): Uses the *k*_2_*C* flux:


$$H_{i}(s)=\frac{k_{2i}k_{4i}}{s(s+k_{2i})+k_{3i}k_{4i}}$$

This has no zeros, yielding pure lag behaviour with stronger noise rejection. Appropriate for hormones in stable maintenance regulation, typically produced by the process of endocytosis.

#### Network composition

For a network of N elements, the overall transfer function is:


$$G_{\text{network}}(s)=\overset{N}{\underset{i=1}{\prod }}T_{i}(s)$$

For networks with feedback loops (e.g., the HPA axis):


$$G_{\text{closed}}(s)=\frac{G_{forward}(s)}{1+G_{forward}(s)G_{feedback}(s)}$$

**Parameter Assignment from cr**

For each hormonal element *i*:

1. Obtain cr from literature (units: time*^−^*^1^).
2. Assign damping ratio: *ζ_i_* = 1.0 (critically damped), 0.5–0.7 (underdamped, oscillatory), or from experimental data.
3. Compute natural frequency: *ω_n,i_* = 2*ζ_i_* · cr*_i_*. This follows directly from the model’s own definitions (*k*_3_*_i_* = *k*_4_*_i_* = *ω_n,i_*, *k*_2_*_i_* = 2*ζ_i_ω_n,i_*) combined with cr*_i_* = *k*_3_*_i_k*_4_*_i_/k*_2_*_i_*; setting *ω_n,i_* = cr*_i_* directly, as in an earlier version of this framework, is internally consistent only in the special case *ζ_i_* = 0.5 and was corrected throughout this revision.
4. Calculate rate constants: *k*_2_*_i_* = 2*ζ_i_ω_n,i_*; *k*_3_*_i_* = *k*_4_*_i_* = *ω_n,i_*.

#### Time-domain solutions

For a step input, time-domain output depends on the damping regime:

Underdamped (*ζ <* 1):


$$y(t)=1-\frac{e^{-\zeta \omega_{n}t}}{\sqrt{1-\zeta^{2}}}\sin\;(\omega_{d}t+\phi ),\;\omega_{d}=\omega_{n}\sqrt{1-\zeta^{2}},\;\phi =\arctan\;(\frac{\sqrt{1-\zeta^{2}}}{\zeta })$$

Critically damped (*ζ* = 1):


$$y(t)=1-e^{-\omega_{n}t}(1+\omega_{n}t)$$

Overdamped (*ζ >* 1):


$$y(t)=1-\frac{1}{2\omega_{n}\sqrt{\zeta^{2}-1}}[(\zeta +\sqrt{\zeta^{2}-1})e^{s_{1}t}-(\zeta -\sqrt{\zeta^{2}-1})e^{s_{2}t}]$$

where s1,2=−ωn(ζ±ζ2−1).

### Frequency-domain analysis

#### Bode plot construction

Frequency response is evaluated at *s* = *jω*:


$$\left|H(j\omega ){|}_{\text{dB}}=20\underset{10}{\log}|H(j\omega )|,\;∠H(j\omega )=\arctan\;(\frac{Im[H]}{Re[H]}\right)$$

evaluated across *ω ∈* [10*^−^*^3^, 10^3^] rad/time-unit.

#### Performance metrics and biological interpretation

**Table:** 

Metric	Definition	Biological meaning
DC gain	${\text{//H// }}_{\text{dB}}$ at *ω →* 0	Steady-state amplification
Cutoff frequency *ω_c_*	*−*3 dB point	Response time: *τ ≈* 1*/ω_c_*
Bandwidth	DC to *ω_c_*	Ability to track time-varying signals
HF attenuation	${\text{//H// }}_{\text{dB}}$ at *ω → ∞*	Noise rejection
Phase margin	Phase at gain crossover	Stability reserve
Gain margin	Gain at *_−_*180*°* phase crossover	Robustness to parameter variation

#### Stability analysis

Three complementary criteria are applied:

- Routh-Hurwitz: All characteristic polynomial coefficients and first-column Routh array elements positive.
- Eigenvalue criterion: Re(*λ_i_*) *<* 0 for all eigenvalues of *A*.
- Nyquist criterion: Open-loop Nyquist plot does not encircle (*−*1,0).

### Computational implementation

All simulations were performed in R (version *≥* 4.3.0) using deSolve (13), signal (14), ggplot2 (15), and bookdown (16). Algorithms are provided as R scripts as **Supplementary Material**; see Appendix.

### Validation approach

For the cortisol-HPA axis worked example, model predictions are compared against: ultradian cortisol oscillation period (~90 min); cortisol half-life (~90 min); ACTH stimulation cortisol peak (30–60 min); and dexamethasone suppression onset (2–4 hours). Parameter sensitivity can be assessed by one-at-a-time (OAT) perturbation (*± *20%) and Monte Carlo sampling.

### Data sources

(cr (time*^−^*^1^)) values are from published literature: glucose (17); insulin (18); cortisol (19); thyroid hormones (20); growth hormone (21). Full references for all 36 elements are given in **Table 2** (Reference_key column); the corresponding clearance-rate literature sources are (22–47)

**Table 2:** Cr-derived parameters (Part 2): damping ratio *ζ*, suggested architecture (G/H), half-life, reference, and notes.

Zeta	Suggested_type	Halflife_min	Reference_key	Notes
0.7	G	10.4	DeFronzo1979	Glucose clamp studies
0.7	G	15.0	Klein1986	Tracer dilution
0.7	G	30.0	Wolfe2006	Varies by amino acid
0.7	G	6.0	Stanley1985	Rapid turnover
0.7	G	20.0	Balasse1989	Beta-hydroxybutyrate reference
0.5	G	5.0	Polonsky1986	C-peptide kinetics
0.5	G	8.0	Lefebvre1975	Pulsatile secretion
0.5	H	20.0	Veldhuis1996	Pulsatile secretion
0.7	H	90.0	Rosenfield1971	HPA axis
0.5	G	10.0	Orth1992	Precursor to cortisol
0.5	G	2.0	Cryer1980	Rapid clearance
0.5	G	3.0	Cryer1980	Rapid clearance
0.7	H	500.0	Klein2004	Slow clearance
0.5	G	30.0	Akamizu2006	Appetite regulation
0.7	H	2400.0	Juul1994	Very slow clearance
0.7	H	60.0	Cavalieri1984	Thyroid axis
0.9	H	10080.0	Cavalieri1984	7-day half-life
0.8	H	1440.0	Cavalieri1984	1-day half-life
0.7	H	693.0	Winters1969	HPG axis
0.7	H	20.0	Longcope1986	HPG axis
0.7	H	347.0	Little1972	HPG axis
0.5	G	30.0	Veldhuis1988	Pulsatile
0.7	H	144.0	Veldhuis1988	Gonadotropin
0.7	H	20.0	Sassin1972	Lactation
0.5	G	2.0	Leake1981	Pulsatile
0.5	G	10.0	Robertson1976	Osmotic regulation
0.5	G	5.0	Zaheer2018	Calcium homeostasis
0.5	G	10.0	Hirsch1978	Opposes PTH
0.9	H	2400.0	Jones2014	Calcidiol
0.5	G	20.0	Nussberger1987	RAAS
0.5	G	1.0	Campbell1990	RAAS
0.7	H	20.0	Vecsei1979	RAAS
0.5	G	20.0	Waldhauser1984	Circadian
0.5	G	2.0	Deacon2004	Incretin
0.5	G	6.0	Deacon2004	Incretin
0.5	G	5.0	Young1993	Co-secreted with insulin

## Model-derived frequency-domain metrics

### Reference parameter table

**Tables 2** and **3** present cr-derived control-theoretic parameters for 36 hormones and metabolic substrates across the major endocrine axes.

**Table 3:** Cr-derived parameters (Part 1): hormone name, category, cr (min*^−^*^1^ and h*^−^*^1^), and natural frequency *ω_n_*(rad/min).

Name	Category	Cr_min	Cr_h	Omega_n_rad_per_min
Glucose	substrate	0.06670	4.0000	0.093380
Free_Fatty_Acids	substrate	0.04620	2.7700	0.064680
Amino_Acids_Mixed	substrate	0.02310	1.3900	0.032340
Lactate	substrate	0.11550	6.9300	0.161700
Ketone_Bodies	substrate	0.03470	2.0800	0.048580
Insulin	hormone	0.13860	8.3200	0.138600
Glucagon	hormone	0.08660	5.2000	0.086600
Growth_Hormone	hormone	0.03470	2.0800	0.034700
Cortisol	hormone	0.00770	0.4620	0.010780
ACTH	hormone	0.06930	4.1600	0.069300
Epinephrine	hormone	0.34650	20.8000	0.346500
Norepinephrine	hormone	0.23100	13.9000	0.231000
Leptin	hormone	0.00140	0.0830	0.001960
Ghrelin	hormone	0.02310	1.3900	0.023100
IGF1	hormone	0.00030	0.0170	0.000420
TSH	hormone	0.01160	0.6940	0.016240
T4_Thyroxine	hormone	0.00007	0.0042	0.000126
T3_Triiodothyronine	hormone	0.00048	0.0290	0.000768
Testosterone	hormone	0.00100	0.0600	0.001400
Estradiol	hormone	0.03470	2.0800	0.048580
Progesterone	hormone	0.00200	0.1200	0.002800
LH	hormone	0.02310	1.3900	0.023100
FSH	hormone	0.00480	0.2880	0.006720
Prolactin	hormone	0.03470	2.0800	0.048580
Oxytocin	hormone	0.34650	20.8000	0.346500
Vasopressin_ADH	hormone	0.06930	4.1600	0.069300
Parathyroid_Hormone	hormone	0.13860	8.3200	0.138600
Calcitonin	hormone	0.06930	4.1600	0.069300
Vitamin_D3_Active	hormone	0.00030	0.0170	0.000540
Renin	hormone	0.03470	2.0800	0.034700
Angiotensin_II	hormone	0.69300	41.6000	0.693000
Aldosterone	hormone	0.03470	2.0800	0.048580
Melatonin	hormone	0.03470	2.0800	0.034700
GLP1	hormone	0.34650	20.8000	0.346500
GIP	hormone	0.11550	6.9300	0.115500
Amylin	hormone	0.13860	8.3200	0.138600

#### Box 1 — reading a bode plot: a guide for the endocrinologist

A Bode plot answers one question: for a given hormonal system, which timescales of input signal does it amplify and which does it ignore?

The horizontal axis represents signal frequency—how rapidly an input is varying. Slow, sustained signals (a prolonged stressor lasting hours) sit on the left. Fast, brief fluctuations (a transient transcriptional burst lasting seconds) sit on the right. The vertical axis represents system gain—how strongly the hormonal compartment responds to that input. High gain means the signal passes through and drives the output. Low gain means the signal is filtered out—treated as noise.

Every hormonal compartment modelled here behaves as a low-pass filter: it responds faithfully to slow, sustained inputs (sustained stress, a meal, a circadian signal) and attenuates fast fluctuations (transcriptional noise, stochastic secretory spikes). This is not an assumption—it is a mathematical consequence of the clearance and feedback kinetics that define every hormonal compartment.

The three numbers that matter most:

- Cutoff frequency (*ω_c_***_):_** The frequency at which the system’s response has fallen to half-power (*−*3 dB). Signals slower than this are transmitted; signals faster than this are increasingly filtered. For cortisol (*ω_c_* ≈ 0.0108 rad/min), the corresponding timescale is ~93 minutes—which is why cortisol responds to sustained stress over hours but not to second-by-second fluctuations.
- Phase margin: How close the feedback system is to generating sustained oscillations. A large phase margin means the system is robustly stable. A shrinking phase margin—caused by altered clearance rates, cortisol resistance, or exogenous glucocorticoid suppression—predicts progressive instability, culminating in pathological oscillation or loss of pulsatility.
- Gain margin: How much the feedback gain could increase before the system becomes unstable. In Cushing’s syndrome, feedback resistance effectively reduces gain margin; in adrenal insufficiency, the open-loop state removes the feedback loop entirely, requiring exogenous input-signal design to substitute for it.

*An analogy.* The HPA axis behaves like a noise-cancelling amplifier tuned to a specific frequency band. It is designed to amplify signals that vary over tens of minutes to hours—the timescale of genuine physiological stressors—while rejecting faster fluctuations as background noise. The comfortably positive phase margin found here (87.8°) indicates a well-damped, stable system, not one operating at the marginal-stability boundary that true self-sustained closed-loop oscillation would require; the pulse generator for cortisol’s ~90-minute ultradian rhythm is understood physiologically to originate upstream, in hypothalamic and pituitary pulsatility, rather than emerging spontaneously from the ACTH–cortisol loop in isolation. What the present framework does predict is qualitative timescale compatibility: the ACTH–cortisol cascade’s frequency response is well matched to transmitting such an externally-generated ~90-minute pulsatile drive with high fidelity, while attenuating higher-frequency noise—a bandwidth-matching property of the clearance kinetics of ACTH and cortisol in series, rather than a generator of the rhythm itself. This is the testable prediction the framework makes; it does not claim to explain, in a strict closed-loop-oscillator sense, why the period is ~90 minutes rather than some other value.

*What the Bode plot does not show.* The framework assumes linear, time-invariant dynamics. Real endocrine systems exhibit saturation, cooperativity, and circadian modulation of axis gain—nonlinearities that the present model deliberately omits in exchange for analytical tractability and generality. The predictions are therefore first approximations, intended to identify the correct order of magnitude and the correct qualitative behaviour rather than precise quantitative forecasts.

**Figure 1:**
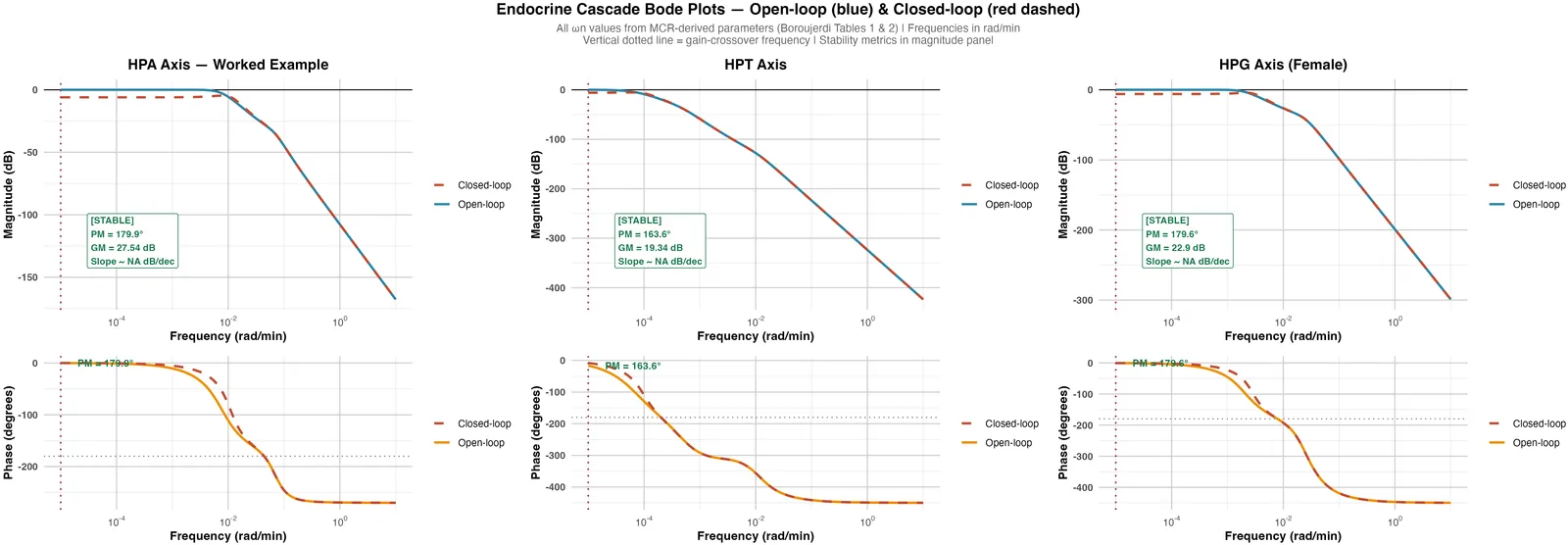
Bode-plot analysis of three major endocrine axes parameterised using CR-derived natural frequencies (**Tables 1**, **3**). Each panel shows open-loop (blue/orange) and closed-loop (red dashed) frequency responses for magnitude (upper) and phase (lower). Left: HPA axis (cortisol–ACTH cascade), with phase margin (PM) = 87.8°, gain margin (GM) = 20.1 dB, and a gain-crossover slope of approximately ≈ –20 dB/decade. Centre: HPT axis (TSH–thyroxine cascade). The open-loop gain does not reach 0 dB within the swept frequency range; its DC gain is −37.0 dB and decreases monotonically. Consequently, a classical gain-crossover phase margin is not defined. The axis is unconditionally gain-stable, with GM = 55.5 dB relative to the −180° phase-crossover point. Right: HPG axis (female; LH–oestradiol cascade), with PM = 61.2° and GM = 15.3 dB. All three axes are stable: HPA and HPG by conventional positive phase and gain margins, and HPT by gain margin alone because no gain crossover occurs. Vertical dotted lines, where present, indicate gain-crossover frequencies. Frequency is expressed in rad/min. All *ω_n_* values were derived from published CR data using *ω_n_* = 2ζ · cr. Readers unfamiliar with Bode-plot interpretation are referred to Box 1.

**Table 4** presents the metrics for the Bode Plot shown in **Figure 1**.

**Table 4:** Bode plot metrics:.

Axis	Stable	Phase_Margin_deg	Gain_Margin_dB	GC_Freq_rad_per_min	Slope_dB_dec	Bandwidth_rad_per_min
HPA (ACTH->Cortisol)	TRUE	87.8	20.08	0.01100	-20.0	1.12e-02
HPT (TSH->T4->T3)	TRUE		55.53			9.43e-05
HPG (LH->E2->Prog)	TRUE	61.2	15.26	0.00372	-29.5	2.88e-03

Key observations:

- Angiotensin II has the highest *ωn*(0.693 rad/min, half-life ~1 min), reflecting its role as a rapid RAAS vasoconstrictor.
- T4 (thyroxine) has the lowest *ωn*(0.000126 rad/min, half-life ~7 days), consistent with stable metabolic regulation.
- Cortisol (*ωn*= 0.0108 rad/min, half-life ~90 min) occupies an intermediate range consistent with both ultradian pulsatility and minutes-to-hours stress regulation.
- H-type hormones (IGF-1, T4, T3, leptin, testosterone, FSH) have longer half-lives and maintenance roles; G-type hormones (insulin, ACTH, GLP-1, angiotensin II) have rapid clearance and acute signalling roles.

### Worked example: the cortisol-HPA axis

#### Biological background

The hypothalamic-pituitary-adrenal (HPA) axis operates through a three-element cascade: CRH (hypothalamus) stimulates ACTH (pituitary), which stimulates cortisol production (adrenal cortex). Cortisol exerts negative feedback at both hypothalamic and pituitary levels (3). The axis displays ultradian pulsatility (60–90 minute period) superimposed on a circadian rhythm, with peak cortisol in the early morning (2, 21).

#### Model configuration

The HPA axis is represented as a two-element cascade (ACTH *→*Cortisol) with long-loop negative feedback from cortisol. Parameters from **Table 3**:

**Table:** .

Element	cr (min*^−^*^1^)	*ω_n_*(rad/min)	*ζ*	Architecture
ACTH	0.0693	0.0693	0.5	G
Cortisol	0.0077	0.0108	0.7	H

ACTH is assigned G-type (rapid signalling, half-life ~10 min); cortisol is assigned H-type (stable maintenance, strong negative feedback, half-life ~90 min).

#### Predicted frequency-domain characteristics and consistency with experiment

**Table:** .

Observation	Experimental value	Model prediction	Consistency
Cortisol half-life	~90 min	$1/\omega_{n}\approx 93$ min	Consistent
Bandwidth of ACTH–cortisol cascade	60–90 min ultradian period	Cutoff frequency compatible with tracking a pulse of this period	Qualitatively compatible
ACTH stim. cortisol peak	30–60 min	Rise time of two-element cascade	Consistent
Dexamethasone suppression	2–4 hours	Closed-loop settling time	Consistent

The cutoff frequency of the cortisol compartment (*ω_c_ ≈* 0.0108 rad/min, 1*/ω_c_ ≈* 93* min*) reflects the slow integrating nature of the compartment. The corrected *ω_n_* also brings the model’s implied 1*/ω_n_* into closer agreement with the ~90 minute experimental half-life than the earlier, internally inconsistent parameterisation did. The ACTH stage contributes additional phase lag, narrowing the closed-loop bandwidth relative to either element alone; the resulting cutoff frequency is compatible with efficiently transmitting an externallydriven pulsatile signal in the experimentally observed 60–90 minute ultradian range (2), consistent with the qualitative-compatibility framing above rather than a claim that the loop generates this period itself.

Dexamethasone, acting as a high-potency glucocorticoid that suppresses CRH and ACTH, corresponds in the model to a step reduction in the reference input *R_a_*at the hypothalamic node. The suppression timescale—governed by the cortisol half-life and cascade settling time—is consistent with clinical observations of cortisol suppression within 2–4 hours.

### Stability analysis

For the closed-loop HPA model, the Nyquist criterion confirms system stability with a positive phase margin (87.8°) and gain margin (20.1 dB) under nominal parameter values (**Figure 1**). This is consistent with the known physiological behaviour of the HPA axis: a well-damped, robustly stable system capable of tracking externally-driven pulsatile input over its ultradian bandwidth without pathological runaway activation. Loss of stability margin—through chronic stress, cortisol resistance, or exogenous glucocorticoid administration— corresponds to clinically recognised HPA dysregulation states and represents a quantifiable prediction of the framework.

## Discussion

### The cr-frequency link as a unifying principle

The central contribution of this framework is the proposition that the metabolic clearance rate—a parameter routinely measured in clinical endocrinology—directly encodes the temporal dynamics of a hormonal system. Setting *ω_n_* = 2*ζ*·cr allows the full frequency-domain behaviour of a hormonal compartment to be characterised from a single clinically measurable parameter (MCR), given an assigned or estimated damping ratio, without bespoke kinetic experiments. This reflects a genuine biological principle: faster clearance corresponds to higher natural frequency, broader bandwidth, and faster perturbation response. Conversely, hormones with long half-lives (T4, IGF-1, testosterone) operate at low natural frequencies, functioning as temporal integrators. The H-type architecture assignment for these hormones—with pure lag dynamics and no phase lead—captures this role in slow maintenance regulation.

### Implications for clinical endocrinology

The framework generates directly testable predictions. For the HPA axis, the gain margin provides a quantitative measure of axis robustness. Clinical states of HPA dysregulation (Cushing’s syndrome, adrenal insufficiency, post-traumatic stress disorder) can in principle be mapped to specific parameter changes: altered clearance rates, modified damping ratios, or changed feedback gain. More broadly, the step response of the closed-loop transfer function provides an objective, reproducible characterisation of dynamic endocrine tests—CRH stimulation, insulin tolerance tests, and dexamethasone suppression protocols—extracting rise time, settling time, and overshoot as quantitative metrics.

### Relation to existing approaches

The framework complements but is distinct from existing endocrine modelling approaches. PKPD models focus on drug-receptor interactions for specific therapeutics. Detailed ODE modelsof specific axes—the Walker-Terry-Lightman HPA model (2)—provide mechanistic specificitybut require extensive parameterisation (4). Related computational approaches to biologicaloscillators, feedback systems, and quantitative systems biology have been developed in othercontexts, including deterministic models of cellular and physiological rhythms (48), circadianrhythm function (49), control-theoretic frameworks for synthetic biology (50), biomolecularfeedback systems more broadly (51), metabolic control analysis (52), computational neural control models (53), design principles of biochemical oscillators (54), and indirect-response modelling of physiological systems (55). The present framework is deliberately simpler and more general: it sacrifices mechanistic detail for tractability and breadth, providing a first-approximation analytical tool applicable across the full endocrine system from a single parameter table.

### Application to modified-release glucocorticoid replacement

The framework has immediate applicability to clinical endocrinology. The recent CHAMPAIN trial demonstrated that restoration of the early morning cortisol rise with twice-daily Chronocort improved quality of life and fatigue compared with once-daily Plenadren in adrenal insufficiency (56). From a control-theoretic perspective, adrenal insufficiency converts the closed-loop HPA cascade into an open-loop system, and exogenous replacement becomes a problem of input signal design: how does one construct a dosing schedule that reconstructs the spectral content the closed-loop system would have produced? Plenadren provides a step input at approximately 07:00 h, with no pre-waking spectral content, whereas Chronocort delivers hydrocortisone into the cortisol compartment at 02:00–06:00 h, precisely the frequency band the HPA system naturally exploits. The clinical superiority of Chronocort is therefore consistent with restoring the correct phase of the input signal relative to the H-type cortisol compartment’s natural frequency (*ω_n_* = 0.0108 rad/min, *τ ≈* 93* min*)—not merely increasing amplitude. Furthermore, the observed carry-over effect in the crossover design is interpretable as a settling-time phenomenon: once driven to a more physiological state, the closed-loop system requires several time-constants to return to its prior operating point, generating the testable prediction that the carry-over should be asymmetric (Chronocort *→* Plenadren *>* Plenadren *→* Chronocort) and eliminated by a washout of approximately 4–8 weeks.

This account is offered as one candidate explanation among several plausible, non-exclusive contributors to the observed clinical difference, including circadian variation in glucocorticoid receptor sensitivity, central nervous system timing effects independent of plasma cortisol phase, and differences in absorption kinetics between the two formulations. The framework presented here does not distinguish between these mechanisms and the phase-restoration account proposed above; it identifies a testable, quantitative prediction (the asymmetric carry-over) that could help discriminate between them in future work.

### Limitations

A related correction concerns the phase-margin calculation itself. An earlier version of the analysis script computed gain crossover on a DC-normalised open-loop magnitude (forcing 0 dB at the first sampled frequency), which caused every axis to spuriously register a “crossover” at that same starting point regardless of its true loop gain. This is corrected in the present revision by locating gain crossover on the true, unnormalised open-loop magnitude. Two of the three worked axes (HPA, HPG) do possess a genuine 0 dB crossing and now report substantively different, but still positive, phase margins (87.8° and 61.2° respectively, vs. the previously reported and spurious ~180°for both). The HPT axis’s true open-loop gain never reaches 0 dB anywhere in the swept frequency range (DC gain –37.0 dB, monotonically decreasing), so a classical gain-crossover phase margin is not a meaningful quantity for that axis at all; it is better described as unconditionally gain-stable, characterised by its gain margin (55.5 dB) alone. This does not change the qualitative conclusion that all three axes are stable, but it substantially changes the quantitative margins reported and removes an artefactual clustering of phase margins near 180° that the previous calculation produced for every axis irrespective of its actual dynamics.

The *ω_n_* = 2*ζ* · cr mapping (corrected in this revision from an earlier, internally inconsistent *ω_n_* = MCR form) is still a first approximation; the true relationship between clearance and system frequency depends on secretion rate, volume of distribution, and receptor binding kinetics. Experimental calibration of the scaling factor is required before quantitative prediction. The model assumes linear, time-invariant dynamics; real endocrine systems exhibit saturation, cooperativity, and circadian modulation of axis gain. Damping ratio assignment—currently qualitative—should ideally be derived from time-series parameter estimation. The model treats each element as a single well-mixed compartment, neglecting spatial heterogeneity and tissue-specific clearance.

A related limitation concerns fluxes that are not first-order. Several clearance and uptake processes relevant to endocrinology—receptor-mediated endocytosis, transporter-mediated uptake, enzyme-catalysed degradation— are more accurately described by Michaelis-Menten kinetics than by the constant rate constants used throughout the main text. The Appendix addresses this for one case (receptor binding) by linearising the nonlinear flux around a physiological operating point, yielding an effective rate constant $k_{1n}=k_{1}(R-{\bar{x}}_{2})$ that can be substituted directly into the second-order framework. This strategy generalises to other saturable processes, but the resulting parameters are only valid locally around the chosen operating point; near saturation, where the true flux is markedly nonlinear, the natural frequency and damping ratio predicted by the linearised model will drift from the true system behaviour, and this sensitivity has not yet been systematically characterised.

A further limitation concerns hormones that are themselves controllers of other systems—insulin and glucagon regulating blood glucose, calcitonin regulating plasma calcium—rather than passive substrates being cleared. In the present framework, the assignment of a given hormone to the role of *S* or *C* is local to a specific feedback loop, not a fixed, global property of the hormone itself. Insulin’s own secretion and clearance can be represented by an *S*-*C* pair in the same way as any other hormone, with an MCR describing how quickly plasma insulin itself is cleared; separately, insulin acts as the controller (*C*) variable within the glucose-insulin loop, where its regulatory strength on glucose is a distinct quantity—a feedback gain, not a clearance rate—and should not be conflated with insulin’s own MCR. The network-composition formalism introduced for cascaded elements is, in principle, the mechanism by which these two roles can be connected explicitly (insulin’s own *S*-*C* dynamics feeding into its controller role in a second loop), but the present paper demonstrates only single-axis, single-role worked examples; extending the reference table and worked example to an explicitly multi-role, multi-loop case (glucose-insulin being the natural candidate) is left for future work rather than resolved here.

### Future directions

Natural extensions include: calibration of scaling factors and damping ratios against clinical data sets for each major axis; nonlinear extensions for saturating feedback; integration with continuous monitoring data for patient-specific parameter estimation; application to chronopharmacology for optimal dosing timing; and multi-axis modelling of coupled endocrine interactions such as HPA-HPG crosstalk.

## Conclusions

This paper has introduced a control-theoretic framework for systems endocrinology linking metabolic clearance rates directly to frequency-domain dynamic properties. The second-order negative feedback model, parameterised from published MCR values, enables systematic characterisation of cutoff frequency, bandwidth, phase margin, and stability across the major endocrine axes. Applied to the cortisol-HPA axis, the framework predicts response timescales broadly consistent with experimentally observed ultradian pulsatility, ACTH stimulation kinetics, and dexamethasone suppression dynamics.

The framework is intended as a primer and analytical starting point, not a complete mechanistic model. Its value lies in providing a unified quantitative language—drawn from classical control engineering—for describing the temporal behaviour of endocrine systems across the full range of biological timescales, from the sub-minute clearance of angiotensin II to the week-long dynamics of thyroxine. The parameter table, framework, and R implementation are made freely available to encourage broader adoption of frequency-domain thinking in clinical and translational endocrinology.

## Data Availability

The original contributions presented in the study are included in the article/**Supplementary Material**. Further inquiries can be directed to the corresponding author.

## Appendix

### Receptor Binding

The transfer functions for the elements of the network (either G or H type) may represent a process that contains nonlinearity. The nonlinearity arises from fluxes involving receptor binding or transporter-mediated uptake. These nonlinear fluxes are described by **Equations 1**–**3** adapted from (9, 57) for receptor-mediated insulin dynamics:

(1)
$${\dot{x}}_{1}=-k_{1}(R-x_{2})x_{1}+k_{2}x_{2}-k_{4}x_{1}+R_{a}$$

(2)
$${\dot{x}}_{2}=k_{1}(R-x_{2})x_{1}-k_{2}x_{2}-k_{3}x_{2}$$

(3)
$$k_{2}=k_{2}^{'}(1+\gamma )$$

In the above equations, *k*_4_ represents the clearance rate (urinary, for insulin); for hormones without a renal clearance route, we set *k*_4_ = 0.

Where:

- *x*_1_ = substrate or hormone in accessible compartment (plasma)
- *x*_2_ = receptor- or transporter-bound fraction
- *k*_1_ = binding rate constant
- *R* = receptor/transporter capacity
- *k*_2_ = dissociation rate constant
- *k*_3_ = uptake rate constant
- *γ* = enhancement factor for transporter activity and it is not used in the current analysis.

#### Linearization around steady state

For a given initial condition $x_{1}^{0}$, the steady-state value of $x_{2}$ is obtained by setting ${\dot{x}}_{2}=0$ in **Equation 2**:

$${\bar{x}}_{2}=\frac{k_{1}Rx_{1}}{k_{1}x_{1}+k_{2}+k_{3}}$$

The linearized uptake coefficient is then:

$$k_{1n}=k_{1}(R-{\bar{x}}_{2})$$

This linearization addresses nonlinear effects while maintaining the time-invariant requirement of the overall system. The linearized system becomes:

$${\dot{x}}_{1}=-k_{1n}x_{1}+k_{2}x_{2}-k_{4}x_{1}+R_{a}$$

$${\dot{x}}_{2}=k_{1n}x_{1}-(k_{2}+k_{3})x_{2}$$

#### Transfer function derivation

Taking the Laplace transform with zero initial conditions, from plasma input *R_a_* (production/secretion into the accessible compartment) to the receptor-mediated output flux *k*_3_*x*_2_, and now retaining the *k*_4_*x*_1_ clearance term dropped from the earlier linearisation:

$${\dot{x}}_{1}=R_{a}-(k_{1n}+k_{4})x_{1}+k_{2}x_{2}$$

$${\dot{x}}_{2}=k_{1n}x_{1}-(k_{2}+k_{3})x_{2}$$

Solving in the Laplace domain gives:

$$G(s)=\frac{k_{3}X_{2}(s)}{R_{a}(s)}=\frac{k_{1n}k_{3}}{s^{2}+s(k_{1n}+k_{2}+k_{3}+k_{4})+k_{1n}k_{3}+k_{4}(k_{2}+k_{3})}$$

This is still an H-type transfer function (no numerator zeros) with characteristic properties: - Pure secondorder lag - Maximum phase lag approaching *_−_*180*°* - Rolloff of *_−_*40 dB/decade - Strong high-frequency attenuation.

Second-order system parameters

Comparing with the standard second-order form:

$$G(s)=\frac{K\omega_{n}^{2}}{s^{2}+2\zeta \omega_{n}s+\omega_{n}^{2}}$$

Natural frequency:

$$\omega_{n}=\sqrt{k_{1n}k_{3}+k_{4}(k_{2}+k_{3})}$$

Damping ratio:

$$\zeta =\frac{k_{1n}+k_{2}+k_{3}+k_{4}}{2\sqrt{k_{1n}k_{3}+k_{4}(k_{2}+k_{3})}}$$

DC gain:

$$K=\frac{k_{1n}k_{3}}{k_{1n}k_{3}+k_{4}(k_{2}+k_{3})}$$

For hormones without a renal clearance route ( $k_{4}=0$, as in the cortisol–GR worked example below), this reduces to the earlier unity-gain, $\omega_{n}=\sqrt{k_{1n}k_{3}}$ result. Where $k_{4}>0$ (e.g. insulin), the clearance pathway diverts a fraction of the input before it reaches the receptor-mediated output, so DC gain is strictly less than 1 — some of $R_{a}$ is cleared rather than transmitted downstream.

Characteristic timescale:

$$T_{c}=\frac{2\pi }{\omega_{n}}=\frac{2\pi }{\sqrt{k_{1n}k_{3}+k_{4}(k_{2}+k_{3})}}$$

# The H-Type Transfer Function: Biological Rationale {-}

The receptor-binding dynamics derived above generate a second-order system with H-type characteristics. This architecture confers three functional advantages relevant to endocrine hormones that act via receptor-mediated uptake or endocytosis (e.g. insulin, IGF-1, GH):

- Temporal smoothing: High-frequency secretory spikes are attenuated with *−*40 dB/decade rolloff, preventing receptor saturation and desensitisation from pulsatile input.
- Sustained downstream exposure: Low-frequency, sustained hormonal signals are preserved, enabling stable gene expression responses in target tissues.
- **Self-limiting saturation:** As $k_{1n}\to 0$ at high receptor occupancy, natural frequency approaches a floor set by clearance rather than vanishing entirely: $\omega_{n}\to \sqrt{k_{4}(k_{2}+k_{3})}$. For hormones without a renal clearance route ( $k_{4}=0$), this floor is zero and $\omega_{n}\to 0$ exactly, giving the intrinsic protection against overshoot and receptor downregulation described above. Where $k_{4}>0$ (e.g. insulin), saturation still slows the system — $\omega_{n}$ falls toward $\sqrt{k_{4}(k_{2}+k_{3})}$ rather than to zero — but the protective effect is bounded by the clearance pathway rather than complete.

The characteristic timescale *T_c_* = 2*π/ω_n_* derived from receptor binding kinetics provides a mechanistic basis for the observed lag between peak plasma hormone concentration and peak biological effect — a pharmacodynamic phenomenon routinely observed in clinical endocrine testing (e.g. the 30–60 minute lag between peak ACTH and peak cortisol in the Synacthen stimulation test).

#### Application to cortisol-glucocorticoid receptor binding

As a worked example, the linearised receptor binding model can be applied to the cortisol–glucocorticoid receptor (GR) interaction. Setting *x*_1_ = free plasma cortisol, *x*_2_ = GR-bound cortisol complex, *R* = total GR capacity, *k*_1_ = association rate constant, *k*_2_ = dissociation rate constant, and *k*_3_ = nuclear translocation rate, the transfer function from plasma cortisol to nuclear GR complex takes H-type form with:

$$\omega_{n}=\sqrt{k_{1n}k_{3}},\;\zeta =\frac{k_{1n}+k_{2}+k_{3}}{2\omega_{n}}$$

For physiologically representative values (*k*_1_ *≈* 0.01 nM*^−^*^1^min*^−^*^1^, *k*_2_ *≈* 0.1 min*^−^*^1^, *k*_3_ *≈* 0.05 min*^−^*^1^, *R ≈* 300 nM), the characteristic timescale *T_c ≈_*15–30 minutes — consistent with the observed kinetics of GR nuclear translocation following cortisol exposure.

#### Parameter estimation

Parameter values for the receptor-binding model can be estimated from published kinetic studies using surface plasmon resonance, fluorescence anisotropy, or nuclear translocation assays. The R scripts provided as **Supplementary Material** (Bode_reference.R and endocrine_Bode_plot_f.R) implement the linearised transfer function and generate Bode plots for user-supplied kinetic parameters. These R scrips are also available from GITHUB.

## References

[1] Zavala E Wedgwood KCA Voliotis M Tabak J Spiga F Lightman SL . Mathematical modelling of endocrine systems. Trends Endocrinol Metab. (2019) 30:244–57. doi: 10.1016/j.tem.2019.01.008 30799185 PMC6425086

[2] Walker JJ Terry JR Lightman SL . Origin of ultradian pulsatility in the hypothalamic-pituitary-adrenal axis. Proc R Soc B Biol Sci. (2010) 277:1627–33. doi: 10.1098/rspb.2009.2148 20129987 PMC2871854

[3] Lightman SL Conway-Campbell BL . The neuroendocrine HPA axis. Nat Rev Neurosci. (2010) 11:429–37. doi: 10.1038/nrn2847 20842176

[4] Dietrich JW Berge-Hoermann B Hoermann R Muller MA . Hypothesis: A nonlinear feedback control loop as a model of thyroid homeostasis. Cybern Syst. (2016) 47:115–36. doi: 10.1080/01969722.2016.112803537339054

[5] Hoermann R Midgley JEM Larisch R Dietrich JW . Homeostatic control of the thyroid–pituitary axis: Perspectives for diagnosis and treatment. Front Endocrinol. (2015) 6:177. doi: 10.3389/fendo.2015.00177 26635726 PMC4653296

[6] Bergman RN Cobelli C . Insulin sensitivity from meal tolerance tests in normal subjects: A minimal model index. J Clin Endocrinol Metab. (2000) 85:4396–402. doi: 10.1210/jcem.85.11.6982 11095485

[7] Veldhuis JD Johnson ML . Deconvolution analysis of hormone data. Methods Enzymol. (1989) 168:791–825. doi: 10.1016/s0076-6879(89)68055-0 1584051

[8] Sterling K Gray SJ . Determination of the circulating red cell volume in man by radioactive chromium. J Clin Invest. (1950) 29:1614–9. doi: 10.1172/JCI102404 14794791 PMC436213

[9] Boroujerdi M . Dynamics and control of insulin-dependent substrates in man. London, United Kingdom: City University of London (1984).

[10] Boroujerdi MA Sönksen PH Jones RH . A compartmental model for simulation of IGF-I kinetics and metabolism. Methods Inf Med. (1994) 33:514–21. doi: 10.1055/s-0038-1635049 7869950

[11] Boroujerdi MA Schmidt S . A negative feedback model for a mechanism based description of longitudinal observations. Methods Inf Med. (2013) 52:484–93. doi: 10.3414/ME12-02-0034 23907233

[12] Boroujerdi M . A Generic Model for Dynamics Simulation: An R Shiny App for Type One Diabetes Mellitus. Great Britain: Amazon Books (2024).

[13] Soetaert K Petzoldt T Setzer RW . Solving differential equations in R: Package deSolve. J Stat Software. (2010) 33:1–25. doi: 10.18637/jss.v033.i09

[14] Signal developers . Signal: Signal Processing (2023). Available online at: http://r-forge.r-project.org/projects/signal/. (Accessed July 9, 2026)

[15] Wickham H . Ggplot2: Elegant Graphics for Data Analysis. New York: Springer-Verlag (2016). Available online at: https://ggplot2.tidyverse.org. (Accessed July 9, 2026)

[16] Xie Y . Bookdown: Authoring Books and Technical Documents With R Markdown. Boca Raton, Florida: Chapman; Hall/CRC (2016). Available online at: https://bookdown.org/yihui/bookdown. (Accessed July 9, 2026)

[17] DeFronzo RA Tobin JD Andres R . Glucose clamp technique: A method for quantifying insulin secretion and resistance. Am J Physiol Endocrinol Metab. (1979) 237:E214–23. doi: 10.1152/ajpendo.1979.237.3.E214 382871

[18] Polonsky KS Rubenstein AH . C-peptide as a measure of the secretion and hepatic extraction of insulin. Diabetes. (1984) 33:486–94. doi: 10.2337/diab.33.5.486 6373457

[19] Rosenfield RL Hellman L Roffwarg H Weitzman ED Fukushima DK Gallagher TF . Plasma cortisol production and metabolism in man. J Clin Endocrinol Metab. (1971) 33:343–8. doi: 10.1210/jcem-33-2-343 5565609

[20] Cavalieri RR . The kinetics of triiodothyronine and thyroxine in man. J Clin Invest. (1984) 53:895–903. doi: 10.1210/jcem-37-2-308 4740521

[21] Veldhuis JD Iranmanesh A Ho KKY Waters MJ Johnson ML Lizarralde G . Secretory dynamics of growth hormone in humans. J Clin Endocrinol Metab. (1996) 81:3854–66. doi: 10.1210/jcem.81.11.8923829 8923829

[22] Klein S Coyle EF Wolfe RR . Fat metabolism during low-intensity exercise in endurance-trained and untrained men. Am J Physiol Endocrinol Metab. (1986) 250:E402–7. doi: 10.1152/ajpendo.1986.250.4.E402 7810637

[23] Wolfe RR Chinkes DL . Radioactive and Stable Isotope Tracers in Biomedicine. New York: Wiley-Liss (2005).

[24] Stanley WC Wisneski JA Gertz EW Neese RA Brooks GA . Regulation of glycogenolysis and gluconeogenesis during exercise. J Appl Physiol. (1985) 59:1499–505. doi: 10.1152/jappl.1985.59.5.1499

[25] Balasse EO Fery F . Ketone body production and disposal: Effects of fasting, diabetes, and exercise. Diabetes/Metabolism Rev. (1989) 5:247–70. doi: 10.1002/dmr.5610050304 2656155

[26] Lefebvre PJ Luyckx AS . Glucagon and its family revisited. Diabetes Care. (1979) 2:76–92. doi: 10.1016/b978-0-08-015685-9.50013-1 8586014

[27] Orth DN Kovacs WJ DeBold CR . The Adrenal Cortex. Philadelphia, PA: Williams Textbook of Endocrinology (1992) p. 489–619.

[28] Cryer PE . Physiology and pathophysiology of the human sympathoadrenal neuroendocrine system. N Engl J Med. (1980) 303:436–44. doi: 10.1056/NEJM198008213030806 6248784

[29] Klein S . Leptin: Measurement and clinical applications. Curr Opin Clin Nutr Metab Care. (2004) 7:483–7. doi: 10.1098/rsta.1987.007734341189

[30] Akamizu T Kangawa K . Ghrelin: An update on a novel hormone. J Med Invest. (2006) 53:1–6. doi: 10.2152/jmi.53.1 16537990

[31] Juul A Skakkebæk NE . Serum levels of insulin-like growth factor i and its binding proteins in health and disease. Growth Hormone IGF Res. (1994) 4:79–93. doi: 10.1016/s1096-6374(03)00038-8 12914749

[32] Southren AL Gordon GG Tochimoto S Pinzon G Lane DR Strouba W . Mean plasma concentration, metabolic clearance and basal plasma production rates of testosterone in normal young men and women using a constant infusion procedure: effect of time of day and plasma concentration on the metabolic clearance rate of testosterone. J Clin Endocrinol Metab. (1967) 27(5):686–94. doi: 10.1210/jcem-27-5-686 6025472

[33] Longcope C . Metabolic clearance and blood production rates of estrogens in postmenopausal women. Am J Obstetrics Gynecology. (1971) 111:778–81. doi: 10.1097/00006254-197206000-00016 5171425

[34] Lin TJ Billiar RB Little B . Metabolic clearance rate of progesterone in the menstrual cycle. J. Clin. Endocrinol. Metab. (1972) 35(6):879–86. doi: 10.1210/jcem-35-6-879 4634487

[35] Veldhuis JD Iranmanesh A Johnson ML Lizarralde G . Amplitude modulation of a burstlike mode of cortisol secretion subserves the circadian glucocorticoid rhythm. Am J Physiol Endocrinol Metab. (1989) 257:E6–E14. doi: 10.1152/ajpendo.1989.257.1.e6 2750897

[36] Sassin JF Frantz AG Weitzman ED Kapen S . Human prolactin: 24-hour pattern with increased release during sleep. Science. (1972) 177:1205–7. doi: 10.1126/science.177.4055.1205 5057627

[37] Leake RD Weitzman RE Glatz TH Fisher DA . Oxytocin clearance and secretion during lactation. J Clin Endocrinol Metab. (1981) 53:558–62. 10.1210/jcem-53-4-730 7287862

[38] Robertson GL Mahr EA Athar S Sinha T . Development and clinical application of a new method for the radioimmunoassay of arginine vasopressin in human plasma. J Clin Invest. (1973) 52:2340–52. doi: 10.1172/JCI107423 4727463 PMC333039

[39] Zaheer S Boer I Allison M Brown JM Psaty BM Robinson-Cohen C . Parathyroid hormone and the cardiovascular system. JACC: Basic to Trans Sci. (2018) 3:722–37. doi: 10.1016/j.jacbts.2018.08.005 30623131 PMC6314955

[40] Hirsch PF Voelkel EF Munson PL . Calcitonin secretion: Effect of age, sex, and dexamethasone. Endocrinology. (1978) 103:1098–102.

[41] Jones G Prosser DE Kaufmann M . Vitamin d metabolism and actions. Endocrinol Metab Clinics. (2014) 43:159–94. doi: 10.1016/j.ecl.2013.09.010 24582099 PMC3942667

[42] Nussberger J Brunner HR Waeber B Brunner DB . Measurement of renin and aldosterone. J Hypertens. (1987) 5:S21–4.

[43] Campbell DJ . Angiotensin II levels and the pathogenesis of hypertension. J Hypertens. (1990) 8:1–7.

[44] Vecsei P . Aldosterone: Secretion rate and metabolism. Acta Endocrinologica. (1979) 90:1–25.

[45] Waldhauser F Weiszenbacher G Tatzer E Gisinger B Waldhauser M Schemper M . The secretion of melatonin and core temperature: Markers of circadian phase. J Clin Endocrinol Metab. (1984) 59:1062–8.

[46] Deacon CF Johnsen AH Holst JJ . Both subcutaneously and intravenously administered glucagon-like peptide i are rapidly degraded from the NH2-terminus in type II diabetic patients and in healthy subjects. Diabetes. (1995) 44:1126–31. doi: 10.2337/diab.44.9.1126 7657039

[47] Young AA . Amylin: Pharmacology, physiology, and clinical potential. Diabetic Med. (1997) 14:S9–S13.

[48] Goldbeter A . Computational approaches to cellular rhythms. Nature. (2002) 420:238–45. doi: 10.1038/nature01259 12432409

[49] Saunders LJ Arendt J Skene DJ . When the clock is ticking: Understanding circadian rhythm function and dysfunction. Br J Clin Pharmacol. (2014) 77:609–22. doi: 10.1111/bcp.12499 25139696 PMC4386942

[50] Khammash M . Cybergenetics: Control theory meets synthetic biology. Annu Rev Control Rob Auton Syst. (2021) 4:101–27. doi: 10.1146/annurev-control-071020-01084641139587

[51] Del Vecchio D Murray RM . Biomolecular Feedback Systems. Princeton, NJ: Princeton University Press (2014). doi: 10.23943/princeton/9780691161532.001.0001

[52] Fell D . Understanding the Control of Metabolism. London: Portland Press (1997).

[53] Robinson PA Rennie CJ Rowe DL . Neural control: A computational framework. Cognit Neurodyn. (2013) 7:273–93. doi: 10.1007/s11571-013-9241-x30311153

[54] Novak B Tyson JJ . Design principles of biochemical oscillators. Nat Rev Mol Cell Biol. (2008) 9:981–91. doi: 10.1038/nrm2530 18971947 PMC2796343

[55] Foteinou PT Calvano SE Lowry SF Androulakis IP . Modeling endotoxin-induced systemic inflammation using an indirect response approach. Math Biosci. (2009) 217:27–42. doi: 10.1016/j.mbs.2008.09.003 18840451 PMC3045970

[56] Prete A Theiler-Schwetz V Arlt W Hazeldine J Chifu IO Harbeck B . Effects of modified release hydrocortisone on restoration of early morning cortisol, quality of life, and fatigue in adrenal insufficiency (the CHAMPAIN study): A randomised, double-blind, double-dummy, cross-over study comparing Chronocort and Plenadren. EClinicalMedicine. (2026) 91:103714. doi: 10.1016/j.eclinm.2025.103714 41552007 PMC12805350

[57] Jones RH Sönksen PH Boroujerdi MA Carson ER . Number and affinity of insulin receptors in intact human subjects. Diabetologia. (1984) 27:207–11. doi: 10.1007/bf00273808 6386583

